# Management of zygomatico maxillary complex fractures

**DOI:** 10.6026/97320630019120

**Published:** 2023-01-31

**Authors:** Abhishek Singh Payak, Preeti Bhadouria, Alisha Singh, Gopakumar Nair, Rajbir Kaur Randhawa, Sachin Thakur, Ramanpal Singh Makkad

**Affiliations:** 1 Department of Dentistry, MGM Medical College, Indore, Madhya Pradesh, India; 2 Department of Oral Medicine and Radiology, MPCD and RC, Gwalior, Madhya Pradesh, India; 3 Department of Dentistry, SRVRS Medical College, Shivpuri, Madhya Pradesh, India; 4 Department of Oral Medicine and Radiology, KD Dental College, Mathura, Uttar Pradesh, India; 5 Department of Oral and Maxillofacial Surgery, Ahemdabad Dental College and Hospital, Bhadej, Ahemdabad, Gujarat, India; 6 Department of Oral and Maxillofacial Surgery, Modern Dental College and Research Centre, Indore, Madhya Pradesh, India; 7 Department of Oral Medicine and Radiology, New Horizon Dental College and Research Institute, Bilaspur, Chhattisgarh, India

**Keywords:** internal fixation, open reduction, Two-point fixation, Three-point fixation, Zygomatic fracture

## Abstract

Zygomatic bone fractures should be effectively diagnosed and treated because they affect how the face is shaped for both aesthetically and functional reasons. It is possible to compare different surgical techniques and their comorbidities objectively through using outcome quantitative assessments, which call for a treatment programme and long-term follow-up. The purpose of this study was to compare the outcomes of two procedures and the effectiveness of the zygomatic bone following open reduction internal fixation (ORIF) employing two-point fixation and ORIF employing three-point fixation. Two groups of twenty patients each were randomly assigned to. Twenty patients in Group A had ORIF treatment using two-point miniplate fixation technique, and twenty patients in Group B received three-point miniplate fixation treatment. Differences between the two categories were ascertained after they had been evaluated in terms of their advantages and disadvantages. We discovered that the two-point fixation group had the fewest facial complications and neurological side effects. At 1 month follow up, Group B's average radiological evaluation score was 2.47± 0.30, and then at 6 months follow up, it was found out to be 1.87±0.47. A significant statistical distinction between the average radiological evaluations was observed in study participants of Group A at follow up done after one month and six months of procedure. Student's paired t- statistical test was utilized from this statistical analysis. (t = 6.54, P < 0.01). On carrying out follow up after one month of surgery, average neurological assessment score in study participants of group A was found out to be 0.22± 0.42, and then at 6 months follow up, it was 0.61±0.63. The average neurological evaluation score in study participants of Group A on carrying out follow up after one month of surgical procedure and and after six months of surgical procedure months showed a significant statistical distinction when utilizing Student's paired t- statistical test (t = 2.51, P = 0.021).It was determined that the best available rehabilitation for the treatment of zygomaticomaxillary complex fractures is open reduction and internal fixation employing two-point fixation by miniplates.

## Background:

Face is the component of the body that is most exposed, therefore it is prone to trauma. Zygomatic bone fractures should be accurately diagnosed and effectively treated since they affect the facial contour both cosmetically and functionally. Owing to its position, fractures of the zygomatic bone is next only to fractures of nasal bone as the most common fracture of bones of mid face. It accounts for 13percent of total fractures of craniofacial region. [1] Given its conspicuous anatomical position and common name, zygomatic bone fractures are one of the sequel injuries to craniofacial trauma that occur most frequently. The zygomatic bone is in immediate contact with the frontal bone, temporal bone, nasal bone, and maxillary bones. They are collectively considered as Zygomatic Complex. As a result, "Zygomatic Complex Fractures" are when fractures of the zygomatic bones involve surrounding bones in the majority of situations. It rarely occurs that the orbital rim and related components are unaffected during fracture of zygomatic complex. Since the fracture line runs via the infraorbital rim, infraorbital depression, and infraorbital foramen, almost all traumas to the Zygomatic Complex area influence the infraorbital nerve either directly or indirectly. Therefore, a variety of sensory neuropathies are present in zygomatic complex fractures. [2,3,4] Fractures of the zygomatic complex require prompt diagnosis and adequate management because they affect how the face is shaped for both aesthetically and functional reasons. It is possible to compare different surgical techniques and their comorbidities objectively through using outcome quantitative assessments, which call for a treatment programme and long-term follow-up. Therefore, it is of interest to compare between the clinical outcomes of two procedures and the effectiveness of the management of fracture of zygomatic bone treated through process of open reduction with internal fixation with approach of two-point fixation and open reduction with internal fixation with approach of three-point fixation. [5, 6, 7, 8, 9, 10, 11, 12, 13, 14]

## Materials and Methods:

## Information source:

Patients presenting for management of injuries of the zygomatico-maxillary bone assembly were considered as study participants for our prospective study.

##  Data sample:

40 patients who suffered fracture of bone in zygomatico-maxillary bone assembly and were chosen based on the criteria listed below underwent the clinical trial. Before including any participants in the research, informed written consent was sought from each one of them.

##  Inclusion standards:

[1] Zygomatic complicated fracture patients

[2] Study participants who were prepared for follow-up.

## Exclusion standards:

[1] Patients whose health has been impaired and who are not suitable candidates for surgery.

## Sample size:

40 patients with fractures of zygomatico-maxillary bone assembly in total were included in the research. Following inclusion, each case's pertinent data was gathered using a standard form. The participants were fully informed about the research and given an explanation in their own language. The requisite consent was received from the relevant staff. All pre - operative photographic records, intra - operative photographic records were carefully maintained. Once the procedure was completed, photographs were taken post operatively and kept as records. There has been completition of the necessary haematological tests. On the basis of the clinical observations and radiographic observation made about traumatized fronto-zygomatic bone assembly, traumatized infra-orbital rim, the participants were split into Groups 1 and 2 following enrollment in the study. Fixation was performed in the frontozygomatic (FZ) area, the zygomaticomaxillary buttress in Group 1 (twenty patients), and fixation was carried out at the frontozygomatic (FZ) area, area of zygomaticomaxillary buttress, and area of infraorbital rim in Group 2 (twenty patients).

## Surgical procedure:

Nasoendotracheal intubation was carried out while under general anaesthesia. Each patient received a routine cleaning and dressing. For hemostasis, local site injections of two percentage lignocaine and one in 80,000 adrenaline were made. A surgical incision with scalpel was made in the vestibule of buccal muosa in maxilla, and the broken zygoma bone was reduced following the surgical approach advised by Keen's involving either Bristow's elevator or Howarth's periosteal elevator. In study participants who underwent management of trauma with approach of two-point fixation, the shattered region of fronto-zygomatic bone assembly was made visible through surgical lateral brow incision that was followed by attainment of adequate reduction. After identifying the fractured areas, reduced fractured segments were fixed using 4-6 holed miniplates. The thickhness of miniplates was in the range of 1.5 mm to 2 mm at the ZMB region involving two point fixation approaches. The thickhness of miniplates was in the range of 1.5 mm to 2 mm at the FZ area involving two point fixation approaches. Infraorbital margins were exposed by placing asurgical incision in infraorbital or subciliary area in study participants managed with three point approach of fixation. Then fixation was completed (three-point fixation group). Once there was attainment of adequate hemostasis, suturing of the muscle layer, suturing of mucosa, and suturing of skin was carried out with 3-0 vicryl suture, 3-0 black braided silk suture, and 5-0 prolene suture, respectively.

## Intraoperative stability:

The stability of the relocated zygomaticomaxillary fracture was assessed intraoperatively using the digital manual palpation technique to determine whether fixation devices needed to be applied.

## Duration of surgery:

The time period was estimated from the time the miniplate was adjusted until the final screw was fixed at the fracture location.

## Facial evaluation:

For the purpose of assessing malar asymmetry, frontal radiographic images and bird's eye radiographic images were obtained. The Holmes and Mathews classification method was used to grade malar asymmetry.

## Radiological evaluation:

On the PNS, a horizontal line was created that touches the supraorbital borders. The glabella was touched to create a vertical median line that crossed two maxillary central incisors. The second line of McGregor and Campbell extended from one side's zygomatic arch across the infraorbital borders to the other side's zygomatic arch. On either sides of the horizontal reference line, a reference vertical line perpendicular to horizontal reference line was drawn. It met with medial aspect of boundary of orbit. It was further extended in downward direction to intersect the second line of McGregor and Campbell. The intersection of the two reference lines was designated as the reference point, and measurements were taken horizontally from the vertical middle line to the intersection and vertically from the horizontally placed reference line to the intersection. The discrepancy between these readings on the side of fracture and the normal side was assessed after taking these different measurements on both the right and left sides.

## Neurological evaluation:

[1] Pinprick tests were used to perform this. Preoperatively, one month after surgery, and six months later, an evaluation was conducted.

[2] Paraesthesia not present (score of 0); paraesthesia present (score of 1).

## Mouth opening:

Vernier callipers were used to do this. Preoperatively, one month after surgery, and six months later, the evaluation was completed. Using the side that had not been exposed to trauma as a control, patients' faces were inspected on both sides. Before each test, the patient will receive an explanation of the various testing techniques and see them in action. He or she will close their eyes during the exams. The same investigator will always conduct the tests in the same sequence. Each patient's file will have a record of the findings. Data will be gathered and documented in a Microsoft Excel spreadsheet that is chronological, and SPSS version 15.0 will be used for analysis. For comparison, the chi-square test statistical test, Kruskal Wallis statistical test, and Mann Whitney U statistical test were utilized.

## Results:

## Regarding following factors, the outcomes of two approaches were assessed and compared:

[1] Evaluation of intraoperative stability

[2] Evaluation of duration of surgery

[3] Evaluation of facial outcomes

[4] Evaluation of radiographic outcomes

[5] Evaluation of neurological outcomes

[6] Evaluation of interincisal mouth opening

[7] Evaluation of associated complications.

Surgery took an average of 104.21±7.91 minutes in Group B and 69.91±13.2 minutes in Group A. A statistically significant distinction in the mean surgical length for patients in both groups was discovered utilizing Student's unpaired t-test (t = 7.02, P = 0.0002).At 1 month follow up, Group A's average score after assessment of facial aesthetic was 2.61±0.63, and at 6 months follow up, it was found out to be 1.81±0.59. A statistically significant distinction between the average facial aesthetic score at 1 month follow up and 6 month follow up was discovered utilising Student's paired t-test statistical test (t = 2.76, P = 0.023). At one month, Group B's average facial aesthetic score was 1.81±0.59, and at six months follow up, it was found out to be 2.51± 0.70. No statistically significant distinction between the average value obtained after assessment of facial aesthetics at one month follow up and six month follow up was discovered with the help of Student's paired t-test statistical tests (t = 1.61, P = 0.021). At 1 month follow up, Group A's average radiological evaluation score was 2.32 ±0.70, and then at 6 months follow up, it was found out to be 1.57 ±0.31. A significant statistical distinction between the average radiological evaluations was observed in study participants of Group A at follow up done after one month and six months of procedure. Student's paired t- statistical test was utilized from this statistical analysis. (t = 1.69, P = 0.021). At 1 month follow up, Group B's average radiological evaluation score was 2.47± 0.30, and then at 6 months follow up, it was found out to be 1.87±0.47. A significant statistical distinction between the average radiological evaluations was observed in study participants of Group A at follow up done after one month and six months of procedure. Student's paired t- statistical test was utilized from this statistical analysis. (t = 6.54, P < 0.01). On carrying out follow up after one month of surgery, average neurological assessment score in study participants of group A was found out to be 0.22± 0.42, and then at 6 months follow up, it was 0.61±0.63. The average neurological evaluation score in study participants of Group A on carrying out follow up after one month of surgical procedure and after six months of surgical procedure months showed a significant statistical distinction when utilising Student's paired t- statistical test (t = 2.51, P = 0.021). The average score after neurological assessment among study participants of Group B has been 0.31±0.53 at 1 month and 0.02± 0.31 at 6 months. The average neurological evaluation score in study participants of Group A on carrying out follow up after one month of surgical procedure and after six months of surgical procedure months showed a significant statistical distinction when utilising Student's paired t- statistical test (t = 4.58, P = 0.001).(Table 1, Table 2)

## Discussion:

The main goal of this research was to carry out comparison between the clinical outcomes of two procedures and the effectiveness of the management of fracture of zygomatic bone treated through process of open reduction with internal fixation with approach of two-point fixation and open reduction with internal fixation with approach of three-point fixation. Digital pressure following reduction is used in this type of investigation carried out by Barry CP with associates [15] to establish whether fixation devices are necessary to be used. Both approaches of two-point fixation and as well as three-point fixation in our contextual investigations revealed sufficient intraoperative as well as postoperative stability at the location of fracture .[16] In our study surgery took an average of 104.21±7.91 minutes in Group B and 69.91±13.2 minutes in Group A. A statistically significant distinction in the mean surgical length for patients in both groups was discovered utilizing Student's unpaired t-test (t = 7.02, P = 0.0002). Face is the fraction of the body that is exposed the most to outer environment, making it more vulnerable to injury. Since zygomatic bone fractures impact the facial contour both visually and functionally, it is important to properly diagnose and treat them. Due to its anatomical location, the zygomatic bone is among the most frequently fractured bones of the mid-face, second only to the nasal bone. It represents 13% of all fractures in the craniofacial region. [1] Zygomatic bone fractures are one of the most common sequelae to craniofacial trauma due to its prominent anatomical location and common nomenclature. The frontal bone, temporal bone, nasal bone, and maxillary bone are all in direct contact with the zygomatic bone. They are referred to as the Zygomatic Complex as a whole..As a result, "Zygomatic Complex Fractures" refer to fractures of the zygomatic bones that frequently affect adjacent bones. Rarely, following zygomatic complex fracture, are the orbital rim and associated components undamaged. Nearly all injuries to the Zygomatic Complex area have an impact on the infraorbital nerve either directly or indirectly because the fracture line passes through the infraorbital rim, infraorbital depression, and infraorbital foramen. Therefore, zygomatic complex fractures can have a range of sensory neuropathies. Because they alter the structure of the face for both aesthetic features and functional features, fractures of zygomatic bone require accurate detection and proper management. Utilizing result quantitative measures, it is able to compare various surgical methods and their comorbidities objectively. In our study it was observed that at 1 month follow up, Group A's average score after assessment of facial aesthetic was 2.61±0.63, and at 6 months follow up, it was found out to be 1.81±0.59. A statistically significant distinction between the average facial aesthetic score at 1 month follow up and 6 month follow up was discovered utilising Student's paired t-test statistical test (t = 2.76, P = 0.023). At one month, Group B's average facial aesthetic score was 1.81±0.59, and at six months follow up, it was found out to be 2.51± 0.70. No statistically significant distinction between the average value obtained after assessment of facial aesthetics at one month follow up and six month follow up was discovered with the help of Student's paired t-test statistical tests (t = 1.61, P = 0.021). In a research by Kelly et al. [13] seven individuals (51%) showed signs of symmetric malar prominence, whereas five additional patients (35%) showed signs of minor asymmetry. They noted that treating ZMC complicated fractures with approach involving three-point fixation and tolerant rigid fixation of fractured sections of bone leads to a reduced frequency of complications that are proportionate to the intensity of traumatic injury.[25, 26] Gadkari et al. [16] study strongly suggested, however, that three-point fixation offers superior outcomes and upholds stability of site of fracture in all three dimensional planes . Three-point visualization and fixation led to undesirable consequences in the trial, like postoperative visible scars. In this study on carrying out follow up after one month of surgery, average neurological assessment score in study participants of group A was found out to be 0.22± 0.42, and then at 6 months follow up, it was 0.61±0.63. The average neurological evaluation score in study participants of Group A on carrying out follow up after one month of surgical procedure and after six months of surgical procedure months showed a significant statistical distinction when utilising Student's paired t- statistical test (t = 2.51, P = 0.021). The average score after neurological assessment among study participants of Group B has been 0.31±0.53 at 1 month and 0.02± 0.31 at 6 months. The average neurological evaluation score in study participants of Group A on carrying out follow up after one month of surgical procedure and after six months of surgical procedure months showed a significant statistical distinction when utilising Student's paired t- statistical test (t = 4.58, P = 0.001). These findings are consistent with a research by Kim JH et al.[26] in which paraesthesia was associated with much higher irritation scores than deformity issues, pain issues or trismus issues, with annoyance levels rising across the board. Last but not least, scores for overall satisfaction tended to drop. [23, 24] When contrasted against approach involving two-point fixation group in the current investigation, paraesthesia in the infra orbital area was most prevalent in the study participants managed with approach of three-point fixation. However, Gawande et al. [25] study's revealed that the three-point fixation group had lessened infraorbital feelings. Even if special attention is exercised, this could increase the danger of further injuring the infraorbital nerve, resulting in its compression. In this study at 1 month follow up, Group B's average radiological evaluation score was 2.47± 0.30, and then at 6 months follow up, it was found out to be 1.87±0.47. A significant statistical distinction between the average radiological evaluations was observed in study participants of Group A at follow up done after one month and six months of procedure. Student's paired t- statistical test was utilized for this statistical analysis. (t = 6.54, P < 0.01). On carrying out follow up after one month of surgery, average neurological assessment score in study participants of group A was found out to be 0.22± 0.42, and then at 6 months follow up, it was 0.61±0.63. The average neurological evaluation score in study participants of Group A on carrying out follow up after one month of surgical procedure and and after six months of surgical procedure months showed a significant statistical distinction when utilising Student's paired t- statistical test (t = 2.51, P = 0.021). The results of our investigation are in contrast to those of a study by Gawande et al. [25] which found that study participants who got managed through closed reduction approach without insertion of miniplate experienced higher severity in depression of function infraorbital nerve function after completition of surgical management of fractures of orbitozygomatic complex.[24,25] In three groups, postoperative problems were almost nonexistent. A little infection that appeared after plates were fixed was treated with regular antibiotics. According to a new study by Luck JD[18] published in January 2020, two-point fixation offers a more stable, aesthetically pleasing, and useful solution than three-point approach. Five out of eight trials, according to Sato et al. [19], demonstrated that three-point fixation approach was more effective than two-point fixation approach for treating ZMC fractures. We can therefore draw the conclusion that three-point fixation reduces malar asymmetry in ZMC fractures better than two-point fixation. According to the authors' findings, two-point fixation is a successful management approach for management of non-favorable ZMC fractures in pediatric patients. In the current investigation, just one patient in the three-point fixation group had postoperative infection. Although there are several reasons why postoperative infection rates should be increased, it has been seen that systemic antibiotics taken after surgery, together with good hygiene practices and antibacterial mouthwash, reduce the risk of infection. [20].

## Conclusion:

It was determined that the best available rehabilitation for the treatment of zygomatico maxillary complex fractures is open reduction and internal fixation employing two-point fixation by miniplates.

## Figures and Tables

**Table 1:** Descriptive data for study participants in group A

		Patient 1 to 4	Patient 5 to 8	Patient 9 to 11	Patient 12 to 14	Patient 15 to 17	Patient 18 to 20	Overall Mean ± SD
Duration of surgery (min)		94±1.2	59±1.4	64±1.3	86±1.1	75±1.0	74±1.4	69.91 ± 13.2
Facial aesthetic (months)	1 months	3±0.12	2±0.14	3±0.11	2±0.02	3±0.06	2±0.07	2.61 ± 0.63
	6 months	2±0.01	2±0.04	1±0.02	2±0.03	1±0.02	2±0.32	1.81 ± 0.59.
Radiological assessment (cm) (months)	1 months	2.1±0.32	2.3±0.11	2.3±0.02	2.4±0.04	1.8±0.02	2.6±0.01	2.32 ± 0.70
	6 months	1.2±0.22	1.8±0.09	2±0.03	1.2±0.02	1.1±0.01	1.1±0.02	1.57 ± 0.31
Neurological assessment (months)	1 months	0±0.001	0±0.001	1±0.02	0±0.00	1±0.03	0±0.01	0.22 ± 0.42
	6 months	0±0.01	0±0.02	0±0.02	0±0.01	0±0.03	0±0.02	0.61 ± 0.63

**Table 2:** Descriptive data for participants in group B

		Patient 1 to 4	Patient 5 to 8	Patient 9 to 11	Patient 12 to 14	Patient 15 to 17	Patient 18 to 20	Overall Mean ± SD
Duration of surgery (min)		99±0.21	102±1.12	105±1.34	112±1.10	105±3.21	97±2.11	104.21 ± 7.91
Facial aesthetic (months)	1 months	4±0.23	3±0.21	4±0.34	2±0.12	3±0.11	2±0.10	1.81 ± 0.59.
	6 months	1±0.02	3±0.04	1±0.01	1±0.03	2±0.02	3±0.01	2.51 ± 0.70.
Radiological assessment (cm) (months)	1 months	2.9±0.03	2.2±0.05	1.8±0.02	1.9±0.03	1.2±0.02	2.2±0.04	2.47 ± 0.30
	6 months	1.8±0.01	1.6±0.02	1.5± 0.04	1.6±0.02	1.2±0.03	1.2±0.04	1.87 ± 0.47
Neurological assessment (months)	1 months	1±0.04	1±0.03	1±0.05	1±0.04	0±0.03	1±0.02	0.31 ± 0.53
	6 months	0±0.002	0±0.008	0±0.007	0±0.006	0±0.004	0±0.001	0.02 ± 0.31
